# Development and validation of a cytokine-driven predictive model for COVID-19 severity

**DOI:** 10.1186/s12879-026-12677-0

**Published:** 2026-01-24

**Authors:** Xiuyu Cui, Yingchun He, Bocheng Yu, Fangxia Zou, Jing Wang, Ping Yan, Weidong Zhao

**Affiliations:** 1 Department of Clinical Laboratory, School of Clinical Medicine, Dali University, Dali, China; 2 Department of Clinical Laboratory, First Affiliated Hospital of Dali University, Dali, China; 3 Department of Ultrasound, First Affiliated Hospital of Dali University, Dali, China; 4 Department of Gastroenterology, First Affiliated Hospital of Dali University, Dali, China; 5 Department of Clinical Laboratory, Second Infectious Disease Hospital of Yunnan Province, Dali, China; 6 Immunology Discipline Team, School of Basic Medicine, Dali University, Dali, China

**Keywords:** COVID-19, Cytokines, Predictive model

## Abstract

**Background:**

This study aims to identify key factors influencing the severity of COVID-19 and to develop a predictive model for assessing disease severity.

**Methods:**

A retrospective analysis was conducted on 164 patients with COVID-19 admitted to the First Affiliated Hospital of Dali University from July 2022 to February 2024, and the integrity of electronic medical records and laboratory databases of all included patients was systematically reviewed. Based on established diagnostic criteria for COVID-19 severity, patients were categorized into two groups: mild/ordinary (94 cases) and severe/critical (70 cases). Lasso regression and multivariate logistic regression were employed to identify independent risk factors, and a predictive model was constructed using a column chart. Internal validation of the model was conducted with receiver operating characteristic (ROC) curves, 10-fold cross-validation and calibration curves. The clinical utility of the model was evaluated using decision curve analysis.

**Results:**

Univariate analysis revealed significant differences between the mild/ordinary and severe/critical groups in levels of IL-4, IL-6, IL-8, IL-10, IL-17, IFN-γ, WBC count, neutrophils (N), lymphocytes (L), red blood cells (RBC), hematocrit (HCT), R-CV, R-SD, CRP, PCT, DBI, AST, TP, ALB, urea, creatinine (CREA), uric acid (UA), sodium (Na), magnesium (Mg), calcium (Ca), and total cholesterol (TC) (all *P* < 0.05). Lasso regression identified IL-6, IL-8, IL-17, IFN-γ, N, R-CV, TP, ALB, and urea as predictive variables. Multivariate logistic regression confirmed that IL-6, IL-8, and IL-17 were independent risk factors for COVID-19 severity. The model demonstrated an area under the curve (AUC) of 0.88, with a sensitivity of 74.3% and specificity of 94.7%. The 10-fold AUCs are 0.691, 0.939, 0.657, 1.00, 0.81, 0.797, 0.75, 0.847, 0.969, and 1.00, with an average AUC of 0.856 (95% confidence interval: 0.791–0.922). The calibration curve showed strong agreement between predicted and actual values. Clinical decision curve analysis shows that the model offers optimal clinical utility, with a net benefit higher than alternative strategies when the probability threshold is between 15% and 95%.

**Conclusions:**

IL-6, IL-8, and IL-17 are identified as independent risk factors for the severity of COVID-19. The predictive model based on these factors provides a reliable tool for assessing disease severity in COVID-19 patients.

## Introduction

Coronavirus Disease 2019 (COVID-19) is an infectious illness caused by Severe Acute Respiratory Syndrome Coronavirus 2 (SARS-CoV-2). While it primarily targets the respiratory system, to the virus can also induce damage to other organs, including the intestines, liver, and nervous system [1]. First identification in late 2019, COVID-19 has since spread rapidly worldwide, posing an unprecedented challenge to global public health systems. The clinical manifestations of COVID-19 are highly heterogeneous, with symptom severity ranging from asymptomatic infections to mild, severe and critical cases. Each category requires distinct levels of care and treatment strategies, and is associated with markedly different prognostic outcomes [2, 3].

Early and accurate identification of patients at high risk of developing severe COVID-19 is critical for optimizing the allocation of healthcare resources, enabling timely clinical interventions, and ultimately reducing both morbidity and mortality. Throughout the pandemic, numerous studies have focused on identifying biomarkers, clinical symptoms, and imaging features linked to disease severity. However, due to the complex and multifactorial nature of COVID-19 pathogenesis, no single biomarker or indicator has proven sufficient to reliably predict disease progression.

Cytokines are bioactive regulatory proteins secreted by immune cells during the body’s immune response, and they play a central role in mediating inflammation and immune regulation. Dysregulated cytokine levels have been implicated in the exacerbation of several other coronaviral diseases, including severe acute respiratory syndrome (SARS) and Middle East respiratory syndrome (MERS) [4]. In COVID-19, the “cytokine storm”–a hyperinflammatory state driven by excessive cytokine release-has been strongly associated with disease severity. Multiple studies have demonstrated that an exaggerated immune responses, particularly one characterized by elevated levels of pro-inflammatory cytokines, contributes significantly to disease deterioration and, in some cases, fatal outcomes [5–7].

Against this backdrop, the present study aims to identify key factors influencing COVID-19 severity and develop a comprehensive predictive model. By enhancing the ability to forecast disease progression, this model could support evidence-based clinical decision-making, improve patient outcomes, and facilitate more effective pandemic management strategies.

## Materials and methods

### Participants

This study constitutes a retrospective analysis of clinical data from 164 patients who were admitted to First Affiliated Hospital of Dali University due to COVID-19 between July 2022 and February 2023. All patients were confirmed to have SARS-CoV-2 infection via laboratory nucleic acid testing. Inclusion criteria were as follows: (1) Age ≥ 18 years; (2) fulfillment of the diagnostic criteria outlined in the Diagnosis and Treatment Plan for Novel Coronavirus Pneumonia (Tenth Edition for Trial Implementation) issued by the National Health Commission of the People’s Republic of China [8]; (3)complete clinical data available during hospitalization, enabling the acquisition of required research data; 4、informed written consent signed by the patient or legal representative. Exclusion criteria included: (1) the presence of other serious respiratory diseases, such as active tuberculosis; (2) severe cardiovascular diseases, including malignant arrhythmias, acute myocardial infarction, stroke, or other conditions that could affect prognosis; (3) pregnant or lactating; (4) severe cognitive impairment or mental illness, precluding research cooperation; (5) participation in other intervention measures within the previous month prior to selection. Ethical approval for this study was granted by the Medical Ethics Committee of First Affiliated Hospital of Dali University (NO. 202304008), and all research procedures adhered to the ethical principles of the Helsinki Declaration. Patient personal information was kept strictly confidential and was used solely for scientific research purposes.

### Diagnostic criteria and grouping

Upon hospital admission, the clinical severity of COVID-19 was evaluated according to established diagnostic criteria [9]: (1)mild-predominantly upper respiratory tract infections such as dry throat, sore throat, cough, and fever; (2)moderate-persistent high fever for more than 3 days or (and) symptoms of cough and dyspnea with a respiratory rate (RR) < 30 breaths/minute and an oxygen saturation > 93% at rest; (3)severe-in adults, meeting any of the specified criteria that cannot be attributed to causes other than COVID-19 infection, and in children, meeting any of the criteria for respiratory distress, hypoxemia, or rapid clinical deterioration; (4)critically requiring mechanical ventilation for respiratory failure, shock, or multiple organ failure necessitating ICU monitoring and treatment. Patients were divided into two groups: mild/moderate (94 cases) and severe/critical (70 cases).

### Data acquisition

Data collected included patient age, sex, body mass index, medical history (hypertension, diabetes, smoking, alcohol consumption), vital signs (heart rate, respiration, blood pressure), hospital stay duration, 12 cytokines, and a range of laboratory parameters including white blood cell count (WBC), neutrophil count (Neu), lymphocyte count (LYC), monocyte count (MONO), red blood cell count (RBC), hemoglobin (Hb), platelet count (PLT), C-reactive protein (CRP), procalcitonin (PCT), total bilirubin (TBil), direct bilirubin (DBil), indirect bilirubin (IBil), total protein (TP), albumin (ALB), creatinine (CREA), uric acid (UA), prothrombin time (PT), activated partial thromboplastin time (APTT), thrombin time (TT), and fibrinogen (FIB).

### Statistical analysis

Statistical analysis was performed using SPSS 26.0 and R-4.2.1 statistical software. Quantitative data that followed a normal distribution were represented as mean ± standard deviation and were compared between group using the independent *t*-test. Non-normally distributed quantitative data were expressed as median (interquartile range, M [P25-P75]) and were compared using the Mann Whitney *U* test. Count data were compared between groups using the chi square test or Fisher’s exact probability method. A *P*-value of < 0.05 was considered statistically significant. The primary outcome measure in this study was disease severity among hospitalized COVID-19 patients. Specifically, patients were categorized into mild/moderate and severe/critical groups based on their severity at admission, and subsequent binary statistical analyses were conducted based on this grouping. Lasso regression was used for variable selection, followed by multiple logistic regression modeling to identify independent risk factors. Given the potential collinearity and correlation among independent variables, dimensionality reduction was performed to avoid model overfitting and to screen for risk factors associated with COVID-19 severity. LASSO regression applies an L1 penalty to regression coefficients, automatically shrinking less important or highly correlated coefficients to zero, thereby achieving simultaneous variable selection and parameter estimation. The optimal penalty coefficient λ was determined via 10-fold cross-validation, selecting the λ value (λ-min) that minimized the cross-validation mean squared error or based on the one-standard-error criterion (λ-se). In this study, the λ value corresponding to the smallest error (λ + 1) was chosen as the optimal threshold for screening predictor variables. R-4.2.1 statistical software and relevant packages were used to construct column prediction models. Internal validation was conducted using the bootstrap method (1000 iterations), and the model’s discriminative power was evaluated with the receiver operating characteristic (ROC) curve. The calibration curve and Hosmer Lemeshow goodness-of-fit test were used to assessed the model’s predictive accuracy, and the clinical decision curve was employed to assess its practical utility.

## Results

### Basic demographic characteristic

There was no significant statistical difference between the two groups in terms of basic demographic information and the presence or absence of past medical history (Table 1). The data revealed that the patients in both groups were comparable in terms of age, gender, body mass index (BMI), and vital signs, suggesting that these factors did not play a significant role in the severity of COVID-19.

**Table 1:** Comparison of general information between two groups of patients

Project	Mild/moderate group (*n* = 94)	Severe/critical group (*n* = 70)	Test	*P* value
Age (years)	57.50(45.75,68.25)	59.00(48.75,71.25)	-0.908	0.364
Gender [(%)]			0.075	0.784
male	49(52.10%)	38(54.30%)		
female	45(47.90%)	32(45.70%)		
BMI (kg/m^2^)	23.18 ± 3.80	23.34 ± 3.75	-0.264	0.792
Vital signs				
Pulse (beats/minute)	86.00(80.00,100.00)	91.00(79.75,100.25)	-0.833	0.405
Breathing (breath/minute)	20.00(20.00,21.00)	21.00(20.00,22.00)	-1.925	0.054
Systolic blood pressure (mmHg)	120.50(108.00,133.50)	119.00(110.00,138.00)	-0.264	0.791
Diastolic blood pressure (mmHg)	75.51 ± 11.15	73.36 ± 13.43	1.121	0.264
Medical history [case (%)]				
Hypertension	31(33.00%)	33(47.10%)	3.383	0.066
Diabetes	15(16.00%)	18(25.70%)	2.376	0.123
Smoke	21(22.30%)	24(34.30%)	2.875	0.09
Drink wine	16(17.00%)	18(25.70%)	1.845	0.174

### Comparison of 12 cytokines

The levels of interleukins (IL-4, IL-6, IL-8, IL-10, IL-17), and IFN - γ in the severe/critical group were significantly higher than those in the mild/ moderate group, with statistically significant differences (all *P* values < 0.05) (Table 2). These findings suggest that these cytokines may be important biomarkers for the severity of COVID-19.

**Table 2:** Comparison of 12 cytokines between two groups of patients

Project	Mild/moderate group (*n* = 94)	Severe/critical group (*n* = 70)	Test	*P* value
IL-1β(pg/mL)	3.42(1.28,7.54)	4.00(0.99,8.17)	-0.457	0.648
IL-2(pg/mL)	1.44(1.01,1.64)	1.35(0.94,1.82)	-0.223	0.824
IL-4(pg/mL)	1.35(0.85,1.68)	1.58(1.29,1.76)	-2.082	0.037
IL-5(pg/mL)	2.33(1.75,3.00)	2.63(1.71,3.42)	-1.057	0.29
IL-6(pg/mL)	2.30(1.65,4.41)	28.89(7.01,54.73)	-8.76	<0.001
IL-8(pg/mL)	3.93(0.45,7.56)	26.82(0.94,60.13)	-4.26	<0.001
IL-10(pg/mL)	1.97(1.51,2.44)	2.94(1.77,5.26)	-3.644	<0.001
IL-12P70(pg/mL)	1.68(1.45,1.89)	1.82(1.35,2.44)	-1.327	0.185
IL-17(pg/mL)	5.12(2.69,15.67)	13.08(3.74,26.09)	-2.864	0.004
IFN-α(pg/mL)	1.58(1.34,1.96)	1.77(1.04,3.13)	-0.896	0.37
IFN-γ(pg/mL)	3.36(2.38,5.89)	5.40(3.64,13.15)	-3.712	<0.001
TNF-α(pg/mL)	3.13(1.60,4.33)	2.42(1.23,6.27)	-0.319	0.75

Note: IL-1β: interleukin 1β, IL-2: interleukin 2, IL-4: interleukin 4, IL-5: interleukin 5, IL-6: interleukin 6, IL-8: interleukin 8, IL-10: interleukin 10, IL-12P70: interleukin 12P70, IL-17: interleukin 17, IFN -α: interferon alpha, IFN -γ: interferon gamma, TNF -α: tumor necrosis factor alpha

### Comparison of laboratory indicators

The mild/moderate group exhibited higher levels of RBC, HCT, TP, ALB, Na, Mg, Ca, and TC compared to the severe/critical group, with significant difference (all *P* values < 0.05). Conversely, the severe/critical group had higher levels of WBC, N, L, R-CV, R-SD, CRP, PCT, DBI, AST, UREA, CREA, and UA, also with significant differences (all *P* values < 0.05) (Table 3).

**Table 3:** Comparison of laboratory indicators between two groups of patients

Project	Mild/moderate group (*n* = 94)	Severe/critical group (*n* = 70)	Test	*P* value
WBC(×10^9^/L)	5.08(3.92,7.01)	8.07(4.82,12.04)	-3.99	<0.001
N(×10^9^/L)	3.46(2.25,5.20)	6.48(3.39,9.80)	-4.555	<0.001
L(×10^9^/L)	0.92(0.55,1.31)	1.16(0.75,1.68)	-3.117	0.002
M(×10^9^/L)	0.39(0.22,0.56)	0.45(0.23,0.70)	-1.774	0.076
RBC(×10^12^/L)	4.46 ± 0.61	4.23 ± 0.76	2.151	0.033
HGB(g/L)	138.00(123.00,147.25)	132.00(111.00,146.25)	-1.831	0.067
HCT (%)	41.35(37.60,43.80)	39.15(34.30,43.10)	-2.036	0.042
MCV (fL)	91.55(89.28,94.75)	92.05(88.20,96.05)	-0.043	0.966
MCH (pg)	30.55(29.70,31.80)	30.60(29.90,32.10)	-0.17	0.865
MCHC(g/L)	333.00(328.00,340.25)	332.50(325.50,341.25)	-0.655	0.512
R-CV (%)	12.80(12.20,13.20)	13.20(12.50,14.85)	-2.851	0.004
R-SD (fL)	42.80(40.88,45.35)	44.10(41.60,48.40)	-2.244	0.025
PLT(×10^9^/L)	208.50(163.00,256.25)	204.00(155.00,269.00)	-0.028	0.977
CRP (mg/L)	7.13(2.05,19.18)	21.39(4.56,48.87)	-3.817	<0.001
PCT (ng/mL)	0.04(0.03,0.07)	0.12(0.06,0.52)	-5.067	<0.001
TBI(µmol/L)	8.25(6.18,11.20)	8.85(6.15,11.53)	-0.748	0.454
DBI(µmol/L)	2.95(2.20,4.13)	3.70(2.40,5.30)	-2.064	0.039
IBI(µmol/L)	5.20(3.95,6.45)	4.85(3.80,6.50)	-0.695	0.487
ALT(U/L)	24.00(15.00,37.50)	27.50(17.75,38.25)	-0.941	0.347
ALP(U/L)	75.00(65.75,96.00)	90.00(65.75,114.00)	-1.802	0.072
AST(U/L)	22.50(17.00,33.00)	27.50(21.00,36.00)	-2.086	0.037
TP(g/L)	67.54 ± 6.35	62.27 ± 7.29	4.937	<0.001
ALB(g/L)	36.96 ± 4.44	32.20 ± 5.31	6.247	<0.001
GLB(g/L)	30.58 ± 4.54	30.07 ± 5.23	0.67	0.504
UREA (mmol/L)	4.84(3.83,6.18)	6.27(4.90,9.75)	-4.362	<0.001
CREA(µmol/L)	57.00(49.75,70.25)	70.00(58.00,96.25)	-4.58	<0.001
UA(µmol/L)	266.50(205.00,334.25)	293.50(243.00,364.50)	-2.063	0.039
K(mmol/L)	3.97 ± 0.53	4.03 ± 0.58	-0.726	0.469
Na(mmol/L)	141.00(139.00,142.25)	140.00(137.00,142.00)	-2.104	0.035
Cl(mmol/L)	104.95(102.58,107.00)	103.80(101.05,106.25)	-1.927	0.054
Mg(mmol/L)	0.91(0.85,0.97)	0.89(0.80,0.95)	-2.068	0.039
P(mmol/L)	1.03(0.89,1.21)	1.01(0.78,1.16)	-1.427	0.154
Ca(mmol/L)	2.16(2.07,2.22)	2.08(1.99,2.15)	-3.73	<0.001
TC (mmol/L)	4.64(4.05,5.42)	4.14(3.30,4.77)	-3.129	0.002
TG (mmol/L)	1.25(0.90,1.70)	1.33(1.02,1.73)	-0.743	0.457
PT(s)	12.40(10.90,13.10)	11.85(10.88,13.50)	-0.427	0.669
APTT(s)	33.45(27.08,36.63)	33.70(27.80,38.40)	-1.107	0.268
TT(s)	17.15(16.05,18.10)	16.65(15.50,18.40)	-0.966	0.334
FIB(g/L)	4.73 ± 1.46	5.11 ± 1.80	-1.499	0.136

Note: WBC: white blood cell count, N: neutrophil count, L: lymphocyte count, M: monocyte count, RBC: red blood cell count, HGB: hemoglobin, HCT: hematocrit, MCV: mean corpuscular volume, MCH: mean corpuscular hemoglobin, MCHC: mean corpuscular hemoglobin concentration, R-CV: coefficient of variation of red blood cell volume distribution width, R-SD: standard deviation of red blood cell distribution width, PLT: platelet count, CRP: C-reactive protein, PCT: procalcitonin, TBI: total bilirubin, DBI: direct bilirubin, IBI: indirect bilirubin, ALT: alanine aminotransferase, ALP: alkaline phosphatase, AST: aspartate aminotransferase, TP: total protein, ALB: albumin, GLB: globulin, UREA: urea nitrogen, CREA: creatinine, UA: uric acid, K: potassium ions, Na: sodium ions, CL: chloride ions, Mg: magnesium ions. P: phosphate ions, Ca: calcium ions, TC: total cholesterol, TG: triglycerides, PT: prothrombin time, APTT: activated partial thromboplastin time, TT: thrombin time, FIB: fibrinogen

### Lasso regression analysis of COVID-19 severity

**Fig. 1:**
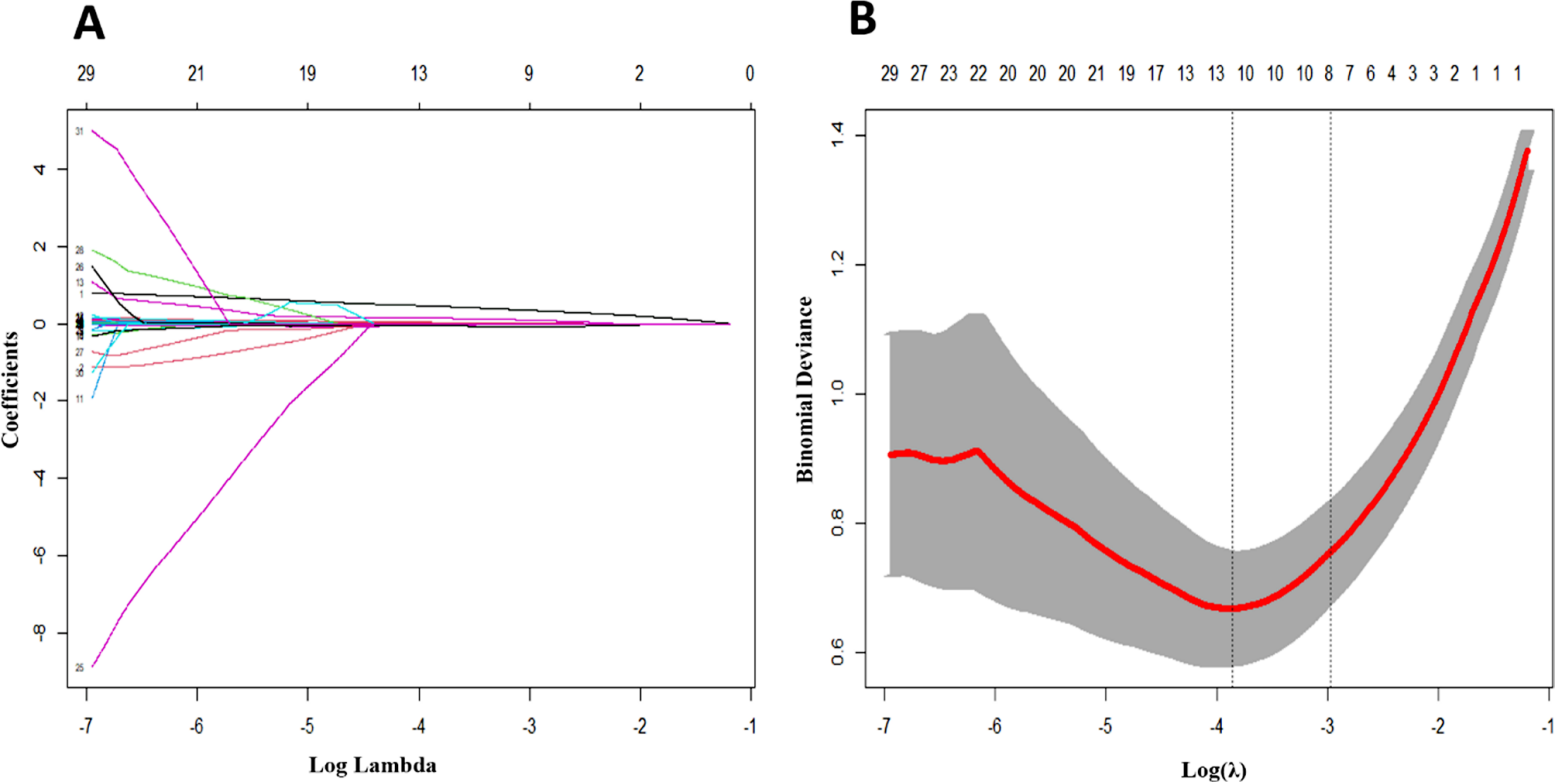
Lasso regression for clinical feature screening. (**A**) shows the dynamic diagram of LASSO regression screening variables, the lower abscissa represents the penalty coefficient, the ordinate represents the regression coefficient, and the upper abscissa represents the number of variables with the non-zero coefficient of the model. (**B**) illustrates the process of selecting the most appropriate predictors by decimal cross-validation in a LASSO model

Lasso regression analysis was performed on 26 variables with *P* < 0.05, identifying IL-6, IL-8, IL-17, IFN-γ, N, R-CV, TP, ALB, and UREA as predictive variables for the severity of COVID-19. The analysis revealed a logarithmic decrease in the number of variables included in the model as the penalty coefficient increased (Fig. 1A). The optimal λ value was determined through 10-fold cross-validation, with the smallest binomial deviation indicating the best fitting effect (Fig. 1B). The final model included the variables IL-6, IL-8, IL-17, IFN-γ, N, R-CV, TP, ALB, and UREA.

### Multivariate logistic regression analysis of the COVID-19 severity

Nine variables selected from the Lasso regression analysis were included in the multivariate logistic regression model. The results indicated that IL-6, IL-8, and IL-17 were independent risk factors for the severity of COVID-19 (all *P* < 0.05) (Table 4). These findings underscore the importance of these cytokines in predicting the severity of the disease.

**Table 4:** Multifactor logistic regression analysis of the severity of COVID-19

Project	βvalue	*P* value	OR	95%CI
IL-6	0.161	0.039	1.041	0.998∽1.085
IL-8	0.035	0.009	1.036	1.009∽1.063
IL-17	0.062	0.045	1.064	1.001∽1.131
IFN-γ	0.001	0.973	1.001	0.95∽1.055
N	0.009	0.931	1.009	0.827∽1.231
R-CV	0.26	0.103	1.297	0.948∽1.775
TP	-0.08	0.273	0.923	0.801∽1.065
ALB	0.014	0.895	1.014	0.821∽1.252
UREA	0.148	0.255	1.16	0.898∽1.497

Note: IL-6: interleukin-6, IL-8: interleukin-8, IL-17: interleukin-17, IFN - γ: interferon - γ, N: neutrophil count, R-CV: coefficient of variation of red blood cell volume distribution width, TP: total protein, ALB: albumin, UREA: urea nitrogen

### Construction of column chart prediction model

**Fig. 2:**
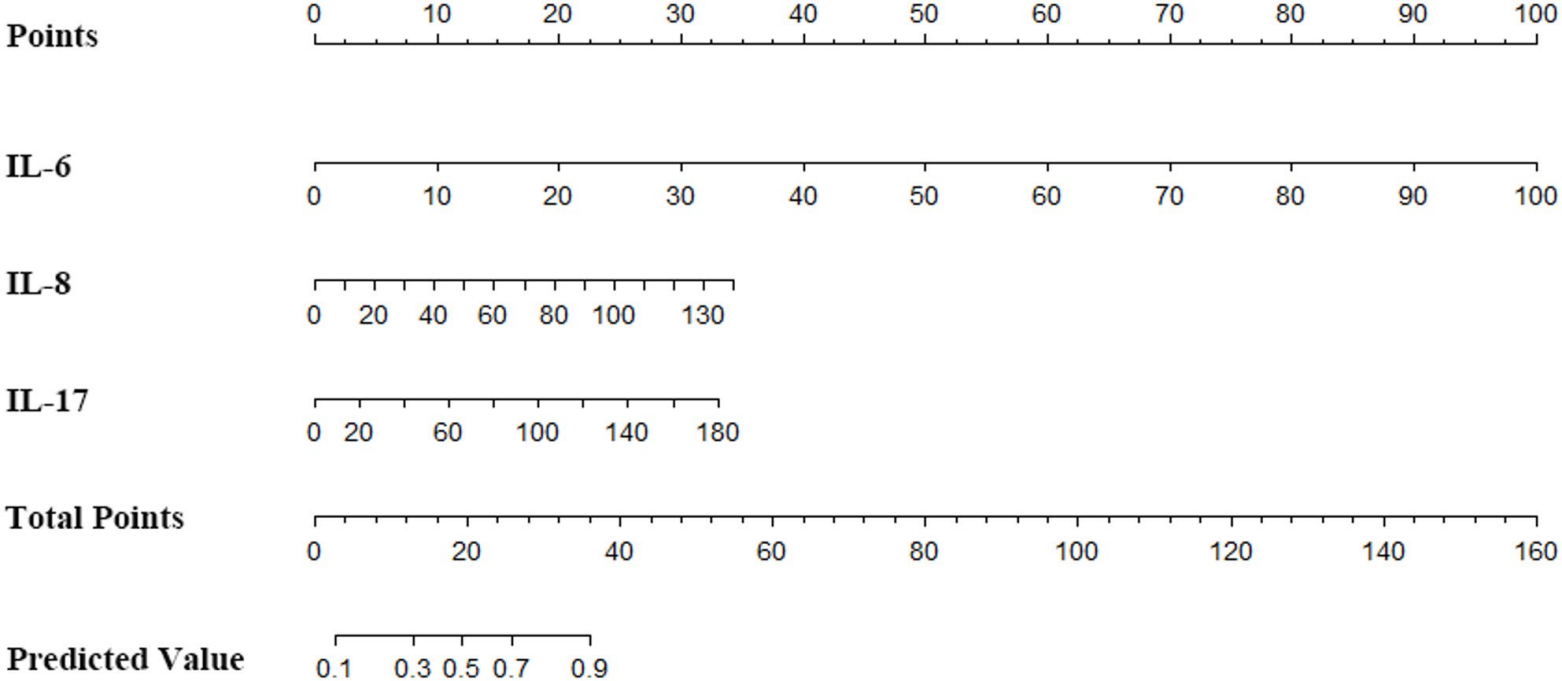
Risk prediction model of COVID-19 severity. Point: Score, which represents the individual score of each variable under different values; IL-6, IL-8, IL-17: the names of the variables in the prediction model, and the scale marked on the line segment corresponding to each variable represents the range of possible values of the variable; Total Points: Total scores, which represent the total scores of the corresponding individual scores of all variables. Predicted Value: The predicted probability of severity risk

A nomogram was constructed incorporating the variables IL-6, IL-8, and IL-17, which were identified as significant predictors of COVID-19 severity (Fig. 2). The nomogram provides a visual tool for assessing the risk of severe disease based on the individual scores of each risk factor.

### ROC curve of predictive models

**Fig. 3:**
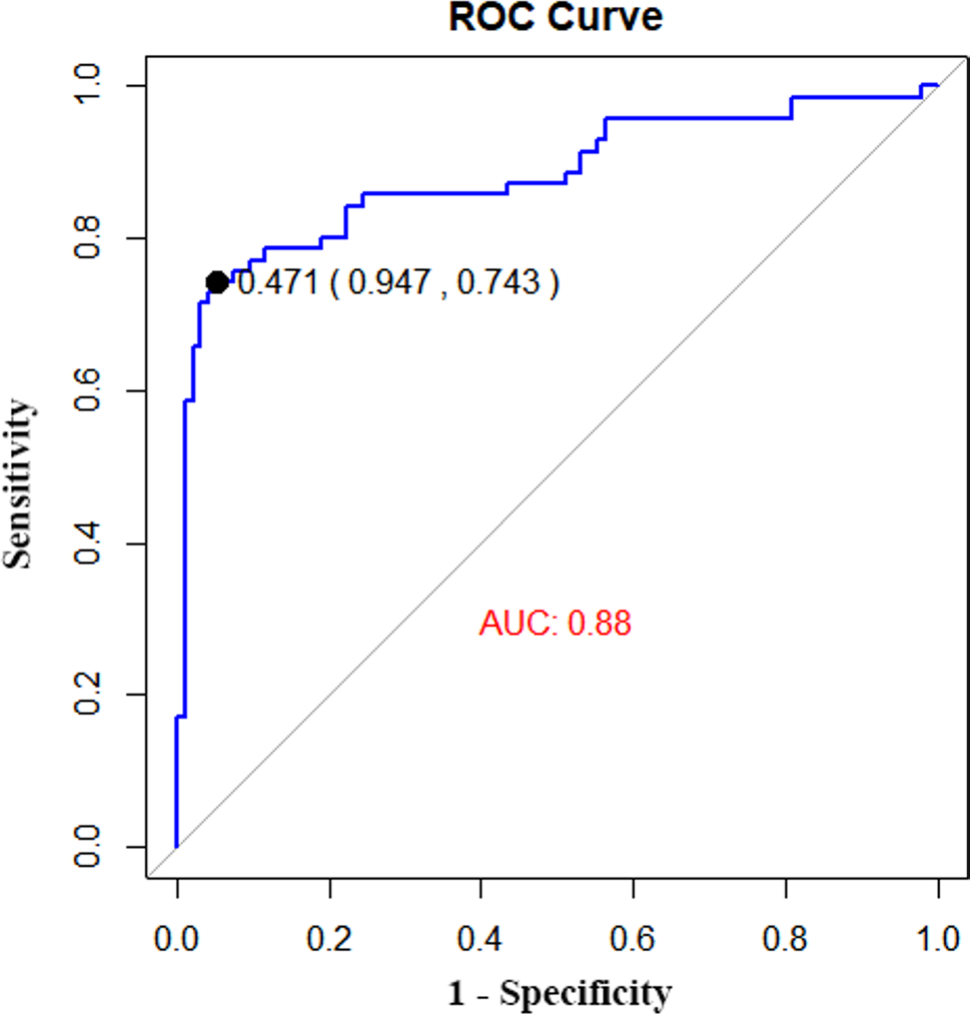
ROC curve of risk prediction model of COVID-19 severity. The abscissa represents the false positive rate (1-specificity), and the ordinate represents the true positive rate (sensitivity). The area of the curve enclosed by the coordinate axis The AUC reflects the magnitude of the diagnostic test value

The predictive model was internally validated using the bootstrap method (1000 iterations), yielding an area under the ROC curve of 0.88. The model demonstrated good sensitivity (74.3%) and specificity (94.7%), suggesting high discriminability (Fig. 3).

### Ten-fold cross-validation of the predictive model

**Fig. 4:**
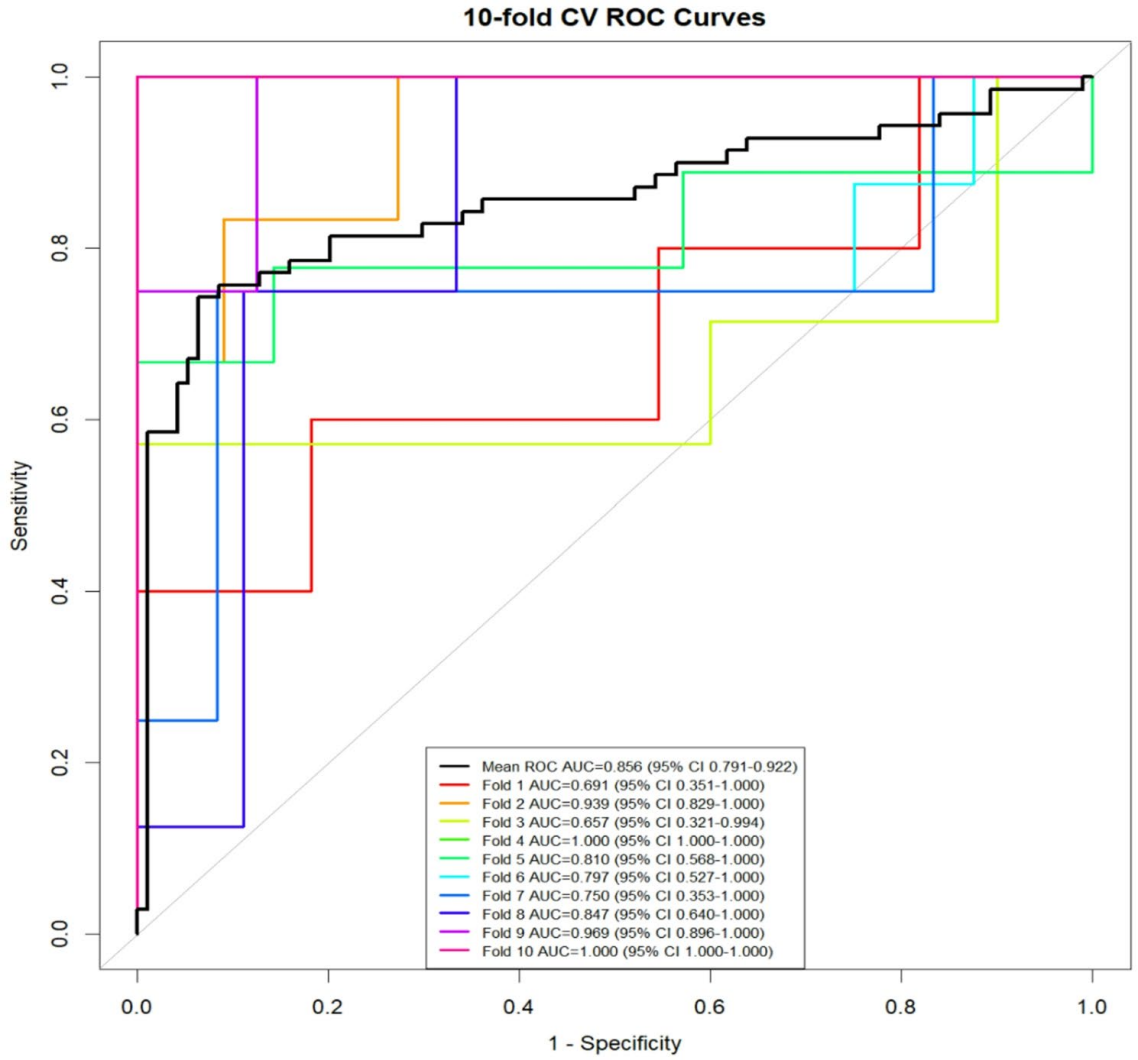
Ten-fold cross-validation curve of the COVID-19 severe risk prediction model. The abscissa represents the false positive rate (1-specificity), and the ordinate represents the true positive rate (sensitivity). The area of the curve enclosed by the coordinate axis The AUC reflects the magnitude of the diagnostic test value. The black line is the ten-fold averaged curve

This study assessed the performance of the predictive model via ten-fold cross-validation. The area under the curve (AUC) values derived from the ten cross-validation folds were 0.691, 0.939, 0.657, 1.00, 0.81, 0.797, 0.75, 0.847, 0.969, and 1.00, with an overall mean AUC of 0.856 (95% confidence interval [CI]: 0.791–0.922). These findings demonstrate that the model possesses favorable discriminative capability. Despite minor fluctuations observed in individual fold results (with the lowest AUC value being 0.657), no evidence of systematic bias was identified (Fig. 4).

### Calibration curve of prediction models

**Fig. 5:**
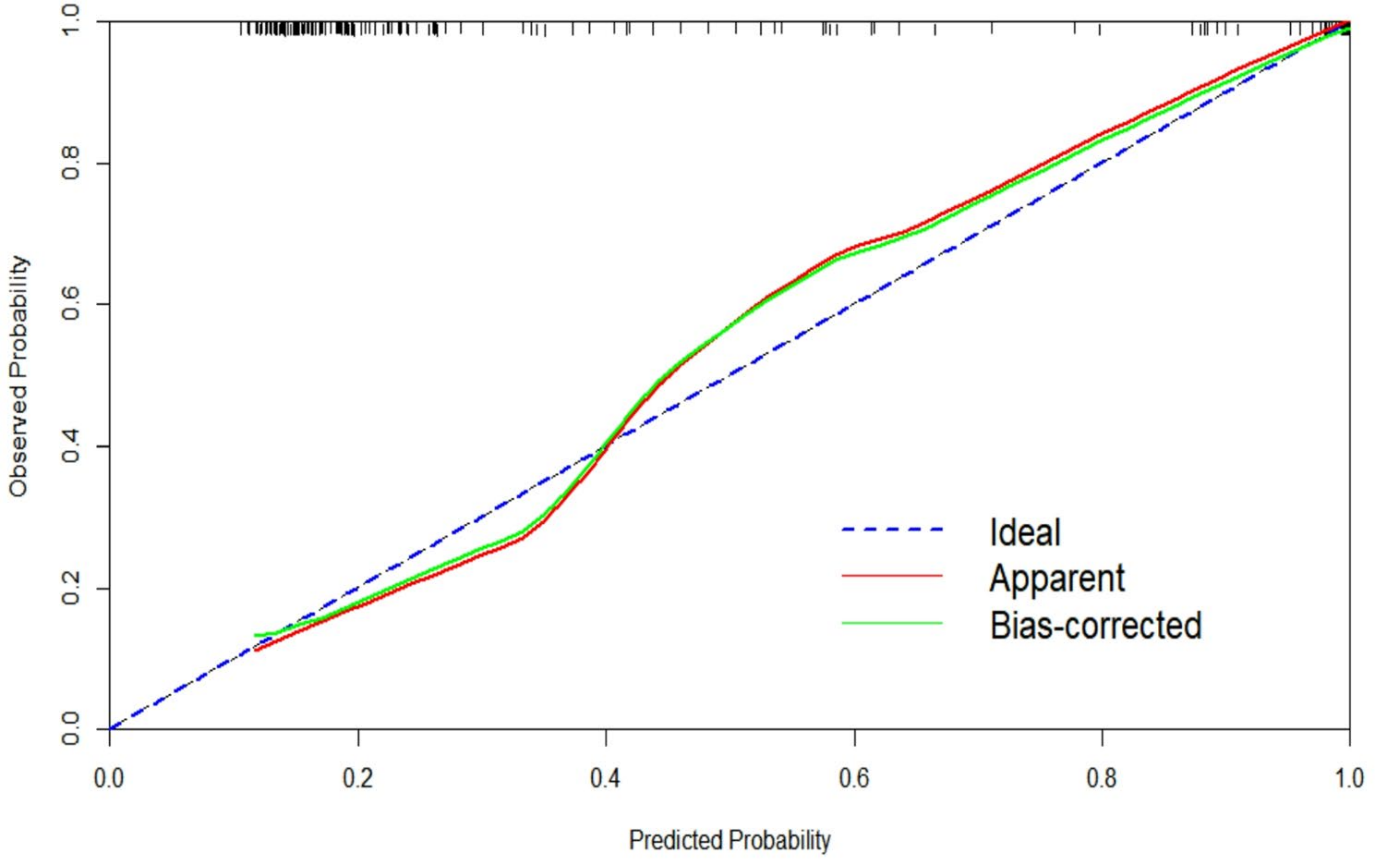
Calibration curve of risk prediction model of COVID-19 severity. The abscissa represents the predicted probability of the event, and the ordinate represents the actual probability of the event. Both coordinates range from 0 to 1. The dotted line in the diagram represents a diagonal reference line, which indicates a situation where the predicted value is equal to the actual value. The red line in the graph represents the fit of the predicted probability with the actual probability. The green line in the graph represents the fit of the corrected predicted probability to the actual probability

The calibration curve of the model was close to the reference curve, indicating good predictive accuracy (Fig. 5).

### Clinical decision curve analysis of predictive models

**Fig. 6:**
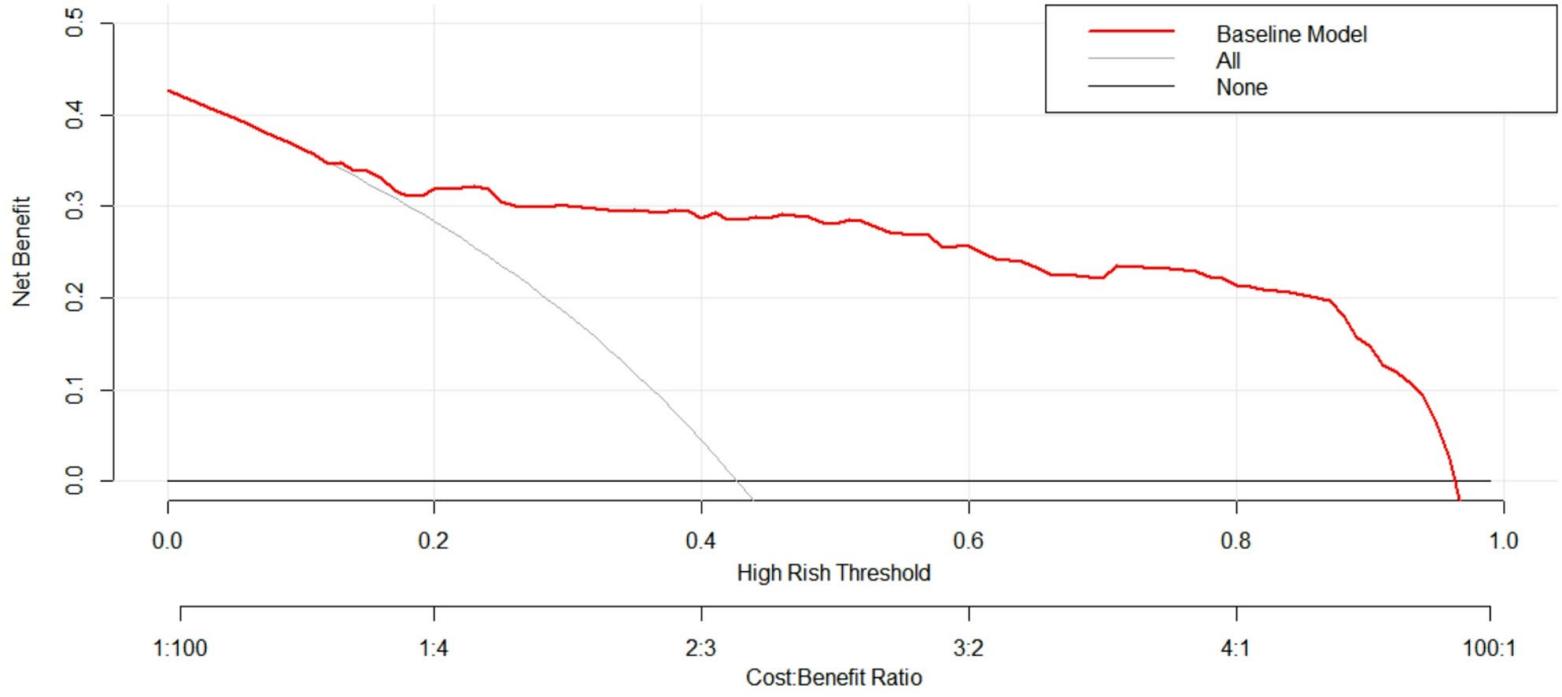
Decision curve of COVID-19 severity risk prediction model. The abscissa is the High Rish Threshold, and the ordinate is the Net benefit. The lower abscissa is the Cost: Benefit Ratio

The clinical decision curve analysis showed that the prediction model had higher net income compared to the two extreme curves when the threshold probability of disease severity was between 15% and 95%, indicating good clinical practicality (Fig. 6).

## Discussion

This study successfully constructed a prediction model for COVID-19 severity using comprehensive clinical data and laboratory indicators. It concluded that the levels of IL-6, IL-8, and IL-17 were significantly elevated in patients with severe/critical COVID-19 compared to those with mild/moderate disease, and these cytokines were identified as independent risk factors for disease severity.

The emergence of COVID-19 posed unprecedented challenges to global health. As research on the disease has advanced, the role of cytokines in its pathogenesis and severity has become a critical area of investigation. Cytokines are a class of biologically active small-molecule proteins synthesized and secreted by immune and certain non-immune cells in response to stimulation. They play a pivotal role in regulating both physiological and pathological immune responses and inflammatory processes [10].

Empirical evidence has linked cytokine storms to the progression of severe and critical COVID-19 cases. This excessive inflammatory response can lead to severe consequences, including increased systemic vascular permeability, tissue edema, multiple organ dysfunction or failure [11, 12]. In patients with severe COVID-19, cytokine storms are often associated with rapid disease progression and poor outcomes. Consistent with this, our findings showed that IL-6, IL-8, and IL-17 levels are significantly higher in the severe/critical group than in the mild/moderate group, confirming their status as independent risk factors for disease severity. These cytokines-released by epithelial or immune cells infected with the novel coronavirus-may exacerbate lung injury by recruiting and activating immune cells, thereby increasing the risk of adverse outcomes. For example, elevated IL-6 levels can directly damage lung tissue, impairing alveolar epithelial and vascular endothelial cells. This leads to diffuse alveolar injury and the development of acute respiratory distress syndrome (ARDS). Additionally, IL-6 promotes thrombosis through multiple pathways, worsening microcirculatory disorders and further damaging the function of other vital organs [13–16].

Novel coronavirus infection activates the immune system, prompting alveolar macrophages, epithelial cells, and other lung cells to secrete large amounts of IL-8. IL-8 levels correlate with the disease stage: they increase in the early stages and rise sharply during severe and critical phases [17–19]. A study analyzing serum samples from COVID-19 patients found that serum IL-8 levels in severe cases were several times higher than in mild cases [20]. Similarly, IL-17 levels are generally elevated in COVID-19 patients, with significant differences observed between severe and mild cases [21, 22].

To translate these findings into clinical practice, our study constructed a nomogram model based on IL-6, IL-8, and IL-17 levels to predict COVID-19 severity. This model exhibits strong discriminative ability, good calibration, and high clinical applicability, providing a valuable tool for the early identification of patients at risk of progressing to severe/critical disease. Compared with traditional clinical scoring systems-such as MELD score for liver failure or the SOFA score for sepsis-our prediction model offers unique advantages. First, unlike general scoring systems like MELD and SOFA, our model uses LASSO regression for data-driven variable selection, enabling more specific prediction of COVID-19 severity. Secondly, in contrast to many complex “black box” machine learning models, our model is presented as a nomogram (bar chart). This design balances high accuracy and clinical interpretability, facilitating its translation into routine clinical practice.

Despite these strengths, our research still has several limitations. (1) It is a single-center retrospective study with a limited sample size, which may introduce selection bias and affect the model’s predictive performance. (2) The nomogram performed well in internal validation (AUC = 0.88), with a mean AUC of 0.856 in ten-fold cross-validation. However, the current evaluation is limited to internal validation-both the training and testing sets were derived from the same preprocessed dataset, ensuring consistent data style and quality. This limitation may lead to insufficient assessment of the model’s generalizability and the risk of overestimating its performance; its robustness to changes in data distribution remains unclear.

3. The model constructed using LASSO regression. While this technique excels at feature selection and handling multicollinearity, it introduces a λ penalty term that shrinks some coefficient to zero. This process can bias the estimates of retained non-zero coefficients, often leading to underestimation of their true effect sizes.

Future research will address these limitations through several directions: conducting multicenter prospective studies to expand the sample size; performing external validation to assess the model’s reliability and applicability, integrating additional biomarkers to improve model accuracy; optimizing machine learning algorithms to enhance performance; and validating the model’s utility in real world clinical settings.

## Conclusion

In conclusion, the COVID-19 severity prediction model developed in this study shows promise for clinical application. However, further refinement and validation are needed to improve its accuracy and reliability. With ongoing optimization, this model could make a significant contribution to the early diagnosis and treatment of COVID-19, enabling more precise medical care for patients.

## Data Availability

The datasets used and/or analyzed during the current study are available from the corresponding author upon reasonable request.
